# The Interaction Between Autophagy and JAK/STAT3 Signaling Pathway in Tumors

**DOI:** 10.3389/fgene.2022.880359

**Published:** 2022-04-26

**Authors:** Jiangyan Xu, Jinrong Zhang, Qi-Fen Mao, Jian Wu, Yuan Wang

**Affiliations:** ^1^ Department of Laboratory Medicine, The First Affiliated Hospital of Zhejiang Chinese Medical University, Hangzhou, China; ^2^ Department of Science and Education, Dafeng District People's Hospital, Yancheng, China; ^3^ Department of Clinical Laboratory, Tongde Hospital of Zhejiang Province, Hangzhou, China; ^4^ Department of Clinical Laboratory, The Affiliated Suzhou Hospital of Nanjing Medical University, Suzhou Municipal Hospital, Gusu School, Nanjing Medical University, Suzhou, China; ^5^ School of Laboratory Medicine, Hangzhou Medical College, Hangzhou, China

**Keywords:** autophagy, JAK/STAT3, interaction, regulation, tumor

## Abstract

Tumor is one of the important factors affecting human life and health in today’s world, and scientists have studied it extensively and deeply, among which autophagy and JAK/STAT3 signaling pathway are two important research directions. The JAK/STAT3 axis is a classical intracellular signaling pathway that assumes a key role in the regulation of cell proliferation, apoptosis, and vascular neogenesis, and its abnormal cell signaling and regulation are closely related to the occurrence and development of tumors. Therefore, the JAK/STAT3 pathway in tumor cells and various stromal cells in their microenvironment is often considered as an effective target for tumor therapy. Autophagy is a process that degrades cytoplasmic proteins and organelles through the lysosomal pathway. It is a fundamental metabolic mechanism for intracellular degradation. The mechanism of action of autophagy is complex and may play different roles at various stages of tumor development. Altered STAT3 expression has been found to be accompanied by the abnormal autophagy activity in many oncological studies, and the two may play a synergistic or antagonistic role in promoting or inhibiting the occurrence and development of tumors. This article reviews the recent advances in autophagy and its interaction with JAK/STAT3 signaling pathway in the pathogenesis, prevention, diagnosis, and treatment of tumors.

## Introduction

Tumor is an ancient disease, which occurs not only in humans but also in plants and animals, and human records of tumors can be traced back thousands of years. The Chinese character “*Liu* (tumor)" was already found in the oracle bone inscriptions of Yin Ruins in China, and the ancient book “*Zhou Li*” (*The Rites of Zhou*), dating 2,400 years ago, recorded that there were doctors specializing in treating swelling and ulcers during the Zhou Dynasty, known as “ulcer doctors”. In the West, there were records of tumors almost at the commence of medical history. In the ancient Egyptian papyrus era, an ointment made of arsenic was applied to treat tumors with ulcers. However, cancer remains a highly fatal disease which has not been conquered by the mankind up to date. According to the Global Cancer Report released by the International Agency for Research on Cancer (IARC), there were 18.1 million new cases of cancer and 9.6 million cancer-related deaths in 2018. The numbers for China were 3.80 million new cases and 2.3 million deaths, ranking first worldwide. Therefore, it is particularly important to investigate the mechanisms, prevention and treatment strategies, and prognoses of tumors.

Tumor had long been regarded as a systemic disease, and it was not until the development of cytopathology that the understanding of tumor histogenesis has been enhanced dramatically. The past century has witnessed the increasingly in-depth research on cancer mechanisms, owing to the development of basic disciplines such as pathology, immunology, and cell biology. The roles of cellular autophagy and JAK/STAT3 signaling pathway in the pathogenesis, development, and treatment of tumors have become hot research topics in recent years (Hu et al., 2020a; Jacquet et al., 2021). Related studies have important significance for the development and prevention of tumor drugs (Khan et al., 2020; Muñoz-Guardiola et al., 2021). This article reviews the recent advances in autophagy and its interaction with JAK/STAT3 signaling pathway in the pathogenesis, prevention, diagnosis, and treatment of tumors.

## JAK/STAT3

**FIGURE 1:**
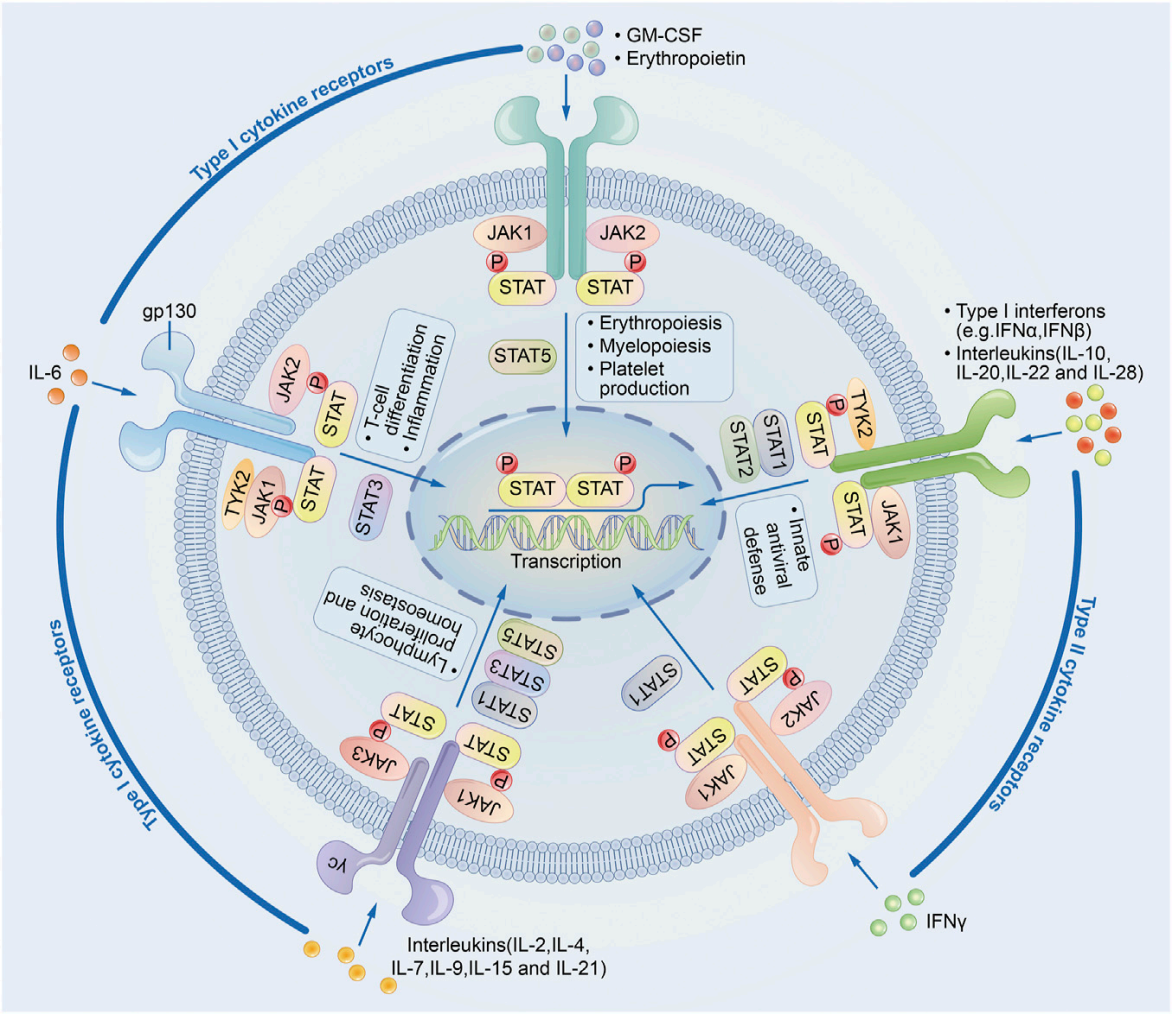
JAK-STAT signaling pathway.

JAK-STAT signal pathway is a cytokine-stimulated signal transduction pathway and is involved in many important biological processes such as cell proliferation, differentiation, apoptosis, and immune regulation. Compared with other signal pathways, JAK-STAT signal pathway is relatively simple. It is composed of three major components: receptor tyrosine kinase (RTK), Janus kinase (JAK), and signal transducer and activator of transcription (STAT) (Figure 1)

JAK has four family members: JAK1, JAK2, JAK3, and Tyk2, which are non-receptor protein tyrosine kinases (PTKs) that play an important role in the signal transduction of cytokine receptor superfamily members (Rodig et al., 1998; Wu et al., 2015). JAKs are the upstream kinases of STAT3, and the phosphorylation of STAT3 is key for its biological activity, so JAKs transmit signals generated by cytokines through the JAK/STAT signaling pathway. Blocking the activity of JAKs can inhibit tumor and inflammation. In a model of acute inflammation of intestinal epithelial cells, JAK-specific siRNAs can significantly inhibit the activity of STAT3, downregulate the expression of IL-1β and TNF-α, inhibit cellular inflammation (Hammarén et al., 2019).

STAT belongs to a unique family of proteins that can bind to DNA. The STAT family includes seven structurally- and functionally-related proteins including STAT1, STAT2, STAT3, STAT4, STAT5a, STAT5b, and STAT6. STAT family members usually consist of 750–900 amino acids. Although the protein structures are similar among these seven members, each STAT protein is encoded by separate genes within the cells (O’shea et al., 2004; Sanda et al., 2013; Furtek et al., 2016; Yu et al., 2009). As a transcription factor, STAT3 plays a key role in signaling and transcriptional activation. Unactivated STAT3 is present in the cytoplasm (Singh et al., 1996); when cells are stimulated, STAT3 binds to and is phosphorylated by specific peptides containing phosphorylated tyrosines through its SH2 and SH3 structural domains (Levy and Lee, 2002). Tyrosine phosphorylation is the classical STAT3 activation pathway and is mainly activated by phosphorylation of a specific tyrosine residue (Tyr705) (Wakahara et al., 2012). STAT3 can be directly catalyzed by either receptor tyrosine kinases (RTKs) (e.g., EGFR, KDR, and MET) or non-receptor tyrosine kinases (e.g., JAKs) (Butti et al., 2018). Once activated, STAT3 polymerizes in the form of activated transcription factors, and these transcriptional activators enter the nucleus to promote transcription of target genes (Kisseleva et al., 2002; Steelman et al., 2004; Wu et al., 2017a).

The JAK/STAT3 axis is a classical intracellular signaling pathway that assumes a key role in the regulation of cell proliferation, apoptosis, and vascular neogenesis, and its abnormal cell signaling and regulation are closely related to the occurrence and development of tumors (Xu and Neamati, 2013; Xiong et al., 2014). It was initially believed that STAT3 was an acute-phase gene transcription enhancer activated by IL-6 (Akira et al., 1994). In subsequent studies, STAT3 was confirmed to regulate many functional genes of cells and bodies, showing inducing or suppressing effects. Its downstream signaling pathways regulate many key molecules (e.g., cyclin D1, survivin Bcl-2, and MMP2) related to cell growth/apoptosis, angiogenesis, and tumor metastasis. For example, STAT3 can stimulate cell proliferation by upregulating Bcl-2; in contrast, STAT3 can also lead to downregulation of MYC and MYB, which ultimately results in cell differentiation and growth arrest (Talbot et al., 2014; Wang et al., 2014; Klupp et al., 2015; Wu et al., 2017b). Many studies have demonstrated that STAT3 is not only involved in the various physiological processes mentioned above but also plays a role in malignant transformation of cells (Yu et al., 2009; Bonetto et al., 2012; Min et al., 2019). Excessive activation of STAT3 has been observed in a variety of tumor cells and tissues. When activated, STAT3 is a point of convergence for many oncogenic tyrosine kinase signaling pathways and displays oncogenicity by promoting cell proliferation and blocking apoptosis through multiple pathways (e.g., EGFR, IL-6/JAK, and Src).

In addition, JAK/STAT3 is also associated with tumor metastasis and blood vessel formation. Hu, F *et al.* confirmed that in colorectal cancer metastasis cells, BECN1 downregulation increased the phosphorylation of STAT3, and that activation of JAK/STAT3 signaling promoted colorectal cancer metastasis (Hu et al., 2020b). In cisplatin-resistant ovarian cancer cells, JAK/STAT3 signaling is hyperactive, with enhanced cell colony-forming capacity and metastasis capacity, which can be reduced by STAT3 inhibitors (Xia et al., 2022). In lung adenocarcinoma cells, highly expressed membrane progesterone receptor α (mPR) activation enhances JAK/STAT3 signaling, increases VEGF secretion in the tumor microenvironment, and promotes tumor blood vessel formation (Xia et al., 2022).

The JAK/STAT3 signaling pathway, as a signaling hub, plays an important role in the tumor epithelial-mesenchymal transformation (EMT). Unno J, *et al.* showed that activated STAT3 binds to the endoplasmic reticulum (ER), induces LIV-1 expression, enhances EMT in prostate cancer (Unno et al., 2009). In cisplatin-resistant lung cancer cells, Ataxia Telangiectasia Mutated (ATM) upregulates the EMT and metastatic capacity of cancer cells by activating the JAK/STAT signaling pathway, thus mediating cell resistance to cisplatin; inhibition of the JAK/STAT pathway by SiRNA significantly reduced the EMT and invasiveness of the resistant cells. The results indicate that JAK/STAT signaling is a mediator of ATM promoting EMT and metastasis in lung cancer cells (Shen et al., 2019).

The role of JAK/STAT signaling pathway in cancer stem cells (CSC) formation is also important. It was found that IL 8-mediated production of IL 6 and TGF 1 promotes the binding of STAT3 to the AUF 1 promoter, reducing p16, p21 and p53 levels, leading to activation of mammary stromal fibroblasts and inducing CSC formation in breast cancer (Al-Khalaf et al., 2019). Studies have found that chemotherapy resistance in myxoid liposarcoma is associated with the number of cells in CSC subsets, and JAK-STAT signaling can control the number of cells with CSC properties. Researchers use the JAK inhibitor ruxolitinib and doxorubicin, which can overcome chemoresistance resistance in MLS patients (Dolatabadi et al., 2019).

Therefore, the JAK/STAT3 pathway in tumor cells and various stromal cells in their microenvironment is often considered as an effective target for tumor therapy. The inhibitors targeting STAT3 are the focus of current research.

The STAT3 inhibitors are usually divided into the following five types. ①: STAT3 DNA binding domain inhibitor: The regulation of gene expression by STAT3 requires the interaction of STAT3 and DNA; therefore, the inhibition of STAT3 DNA binding domain may reduce the activity of STAT3 and intervene in the regulation of gene expression by STAT3. DBD-1 is a small molecule peptide that specifically recognizes the STAT3 DNA binding domain and causes significant increased apoptosis in murine melanoma B16 cells (Nagel-Wolfrum et al., 2004). It confirms that the STAT3 DNA binding domain may be a novel target for anticancer therapy. ②: STAT3 N-terminal domain inhibitors: The STAT3 N-terminal domain has eight helices, which are involved in STAT3 dimerization, assembly of transcriptional complexes, and binding to promoter. It has been demonstrated that STAT3 inhibits the transcription of pro-apoptotic genes in tumor cells through its N-terminal domain (Timofeeva et al., 2013). Thus, inhibitors targeting the STAT3 N-terminal domain may also inhibit tumorigenesis. ③: STAT3 SH2 domain inhibitor: The STAT3 SH2 domain plays an important role in mediating the interaction between receptor tyrosine residues and its dimerization process (Johnston and Grandis, 2011). Thus, inhibition of the SH2 domain not only interferes with the activation of this transcription factor, but also affects its dimerization. For example, garcinol, a polyisoprenylated benzophenone, was reported that can directly bind to the SH2 domain of STAT3 and inhibit its dimerization and acetylation, thus affecting its binding to DNA (Sethi et al., 2014). ④: Inhibition of the STAT3-importin interaction: Importin is a member of a family of nuclear transport receptors. Importin helps in the nuclear translocation of activated STAT3. In colorectal cancer cells, inhibition of the STAT3-importin interaction interferes with cytoplasmic-nuclear displacement, leading to increase in apoptosis (Souissi et al., 2011). Therefore, the elimination of STAT3 nuclear translocation by pharmacological inhibitors could be used as a promising strategy to prevent and treat tumors. ⑤: Blocking the activity of upstream kinases: The biological activity of STAT3 depends on upstream kinase phosphorylation, so the inhibition of upstream kinase activity is a reasonable way to prevent and treat tumos. For example, inhibition of JAK with synthetic compounds such as AZD1480 ultimately inhibits the growth of human melanoma cells and Hodgkin’s lymphoma cells (Scuto et al., 2011). In addition, compounds of natural origin have been shown to have low toxicity profiles; meanwhile, they have synergistic effects with anticancer drugs and may potentially reverse chemotherapy resistance (Cort and Ozben, 2015). Several small-molecule natural STAT3 inhibitors are currently under investigations (Subramaniam et al., 2013; Siveen et al., 2014a; Siveen et al., 2014b). They have shown significant effects in inhibiting STAT3 in a variety of tumor cell lines and preclinical cancer models and may be used as potential anticancer therapeutic agents.

## Autophagy

Autophagy was first observed in rat hepatocytes by Porter and his student Ashford using electron microscopy in 1962 (Shim et al., 2003); Christian de Duve coined the name “autophagy” in 1963 (Kabeya et al., 2000; Zhou, 2016). Autophagy is a process that degrades cytoplasmic proteins and organelles through the lysosomal pathway. It is evolutionarily highly-conserved and is a fundamental metabolic mechanism for intracellular degradation (Mohan et al., 2000). Physiologically, it maintains the dynamic balances between intracellular protein synthesis and degradation and between organelle synthesis and clearance. Therefore, autophagy is often referred to as a recycling process (Cosway and Lovat, 2016). Under pathological conditions, abnormal autophagy often leads to dysfunctional cellular clearance, which in turn triggers the development of a variety of diseases (including neurodegenerative diseases, tumors, muscle diseases, cardiovascular diseases, autoimmune diseases, and tissue fibrosis) by inducing biological effects such as inflammation (Okura and Nakamura, 2012).

The autophagic process is participated by several autophagy-associated proteins (ATGs), which mainly include ULK1/Atg1, ATG5, BECN1/Beclin 1/ATG6, ATG7, LC3, and ATG9A (Mizushima et al., 2011). LC3 exists in two forms, namely cytosolic (LC3-I) or membrane bound (LC3-II). During autophagy, LC3-I in the cytoplasm enzymatically cleaves a small segment of polypeptide and converts them into LC3-II. Therefore, the expression of LC3-II is positively correlated with the level of autophagy and thus can be used to mark the occurrence of autophagy (Klionsky, 2008; Agrotis et al., 2019).

The mechanism of action of autophagy is complex and may play different roles at various stages of tumor development. For example, enhanced autophagic activity may play a role in inhibiting cancer cell proliferation and metastasis during tumorigenesis by clearing the damaged organelles and stabilizing genome; with the progression of the disease, autophagy may promote cancer cell survival and proliferation *via* the following mechanism: the cancer cells initiate the protective autophagy in a hostile environment to degrade nonessential organelles and proteins, thus offering essential energy support for cell survival (Abraham et al., 2018; Akkoç and Gözüaçık, 2018; Bishop and Bradshaw, 2018; Limpert et al., 2018; Wang et al., 2019; Zamame Ramirez et al., 2020). In mice, loss of the ATG gene and other autophagy regulators makes the animals more prone to tumorigenesis (Mariño et al., 2007); in addition, it has been observed that spontaneous tumors are more likely to occur in Becn1 heterozygous mice, which may be related to their reduced autophagy levels *in vivo* (Qu et al., 2003); a similar situation was observed in immortalized epithelial cells lacking Becn1 or ATG5, with increased DNA damage, gene amplification, aneuploidy, and enhanced cell tumorigenicity (Mathew et al., 2009). Similarly, Becn1 allele deletion has also been found in two thirds of patients with breast, ovarian, or prostate cancers (Morikawa et al., 2015).

However, activation of autophagy can help the advanced cancers to cope with intracellular and environmental stresses (e.g. hypoxia, nutritional deficiencies, and cancer treatment), thus favoring tumor progression (Macintosh and Ryan, 2013). Yoshihara *et al.* detected the expression levels of autophagy-related markers (e.g. LC3, p62) in 50 cutaneous SCC specimens, calculated the percentage of positive cells in each low-magnification microscopic field, and assessed their correlation to clinicopathological factors (Yoshihara et al., 2014). The results showed that autophagy was activated during disease progression. As the number of autophagosomes increased, LC3 expression was increased, while p62 expression was decreased. In addition, Sivridis *et al.* applied IHC technology to measure the thickness and LC3 expression of different skin squamous cell carcinoma (SCC) tissue samples. The results showed that LC3 expression was more in the high thickness group (>6 mm), which was significantly different from SCC in the moderate thickness group (2.1–6 mm) and the mild thickness group (<2 mm), This study suggested that high LC3 expression could be used as a marker of tumor invasive ability in SCC tissue (Sivridis et al., 2011).

Although, autophagy provides an important path for cancer cell survival in cases of nutrient deficiency. However, the induction of autophagy depends on the interaction between the concentration of diet-derived nutrients and their respective nutrient sensors. Studies have shown that diet-derived nutrients concentration plays an important role in regulating autophagy induction, and the concentration signal of growth factors, amino acids and low glucose controls the induction of autophagy through ULK1, ATG13 and RB1CC1 protein (Mack et al., 2012; Saxton and Sabatini, 2017).

Membrane type 1 matrix metalloproteinase (MT1-MMP) functions to degrade extracellular proteins and transduce intracellular signals. A series of studies have elucidated the mechanism by which MT1-MMP promotes autophagy and induced autophagic death in tumor cells. MT1-MMP expression was increased in ConA-activated glioblastoma cells, and the expression of autophagy-related markers Bcl-2, ATG9, and LC3 was also enhanced, with autophagosome formation; after silencing of the MT1-MMP genes by SiRNA, the expression of these genes was decreased and autophagy was inhibited (Pratt et al., 2012; Pratt et al., 2016; Desjarlais and Annabi, 2019).

## Interaction Between JAK/STAT3 Signaling Pathway and Autophagy

Many studies have demonstrated that the altered STAT3 expression is accompanied by the abnormal autophagy activity, and they may promote or inhibit the occurrence and development of tumors in a synergistic or antagonistic manner in different tumor stages (Tables 1, 2).

**TABLE 1:** Regulation of tumor autophagy by STAT3.

Author	Cell	Regulation Ways	The Direction of Regulation
Siegelin et al. (Macintosh and Ryan, 2013)	glioblastoma cells	low-concentration sorafenib significantly inhibited cell proliferation and STAT3 phosphorylation and induced apoptosis and autophagy	negatively regulate
You Lk et al. (Yoshihara et al., 2014)	lung cancer cells	Crizotinib induced cytoprotective autophagy by inhibiting STAT3 expression in lung cancer cells, which led to the development of drug resistance.	negatively regulate
Chen et al. (Sivridis et al., 2011)	lung cancer cells	the deletion of cycHIPK3 induced autophagy through the MIR124-3p-STAT3-PRKAA/AMPKa axis	negatively regulate
Tai et al. (Saxton and Sabatini, 2017)	hepatocellular carcinoma cells	led to disruption of the Beclin 1-Mcl-1 complex through downregulation of p-STAT3, which in turn decreased Mcl-1 expression; however, sorafenib did not affect the amount of Beclin 1	negatively regulate
Real et al. (Pratt et al., 2016)	estrogen receptor-negative metastatic breast cancer cells	activation of the STAT3-Bcl-2 pathway inhibited the autophagy	negatively regulate
Qin et al. (Desjarlais and Annabi, 2019)	lymphoma U937 cells	IL-6 inhibits cellular autophagy through activation of the STAT3 signaling pathway.	negatively regulate
Cao et al. (Kroemer et al., 2010)	gastric cancer cells	CYT997 induce autophagy and apoptosis in gastric cancer cells by activating mitochondrial ROS accumulation and silencing the JAK2/STAT3 pathway	negatively regulate
Blessing et al. (Maycotte et al., 2014)	ovarian cancer cells	Crizotinib induced autophagy and mediated apoptosis in cancer cells by reducing the phosphorylation of STAT3 and Bcl-2 expression	negatively regulate
Zhang et al. (Yokoyama et al., 2007)	colorectal cancer cells	GRIM19 inhibits autophagy by inactivating the STAT3/HIF‐1α signaling axis	negatively regulate
Yamada et al. (Liu et al., 2018)	pancreatic cancer cells	the activated IL-6/STAT3 pathway upregulated autophagy levels in pancreatic cancer cells.	positively regulate
Pratt et al. (Rouschop et al., 2010)	glioblastoma U87 cells	ConA induced overexpression of membrane-type 1 matrix metalloproteinase (MT1-MMP) gene and protein, followed by increased STAT3 phosphorylation, which finally led to increased autophagy	positively regulate
Yang et al. (You et al., 2015a)	gallbladder cancer cells	the kinase activator MOB1A plays a key role in the development of gallbladder cancer (GBC) by promoting autophagy through activation of the IL6/STAT3 signaling pathway	positively regulate

**TABLE 2:** Regulation of STAT3 by autophagy.

Author	Cell	Regulation Ways	The Direction of Regulation
Shi et al. (Chen et al., 2020)	macrophages and lymphocytes	Inhibition of autophagy by 3-methyladenine (3-MA), a specific autophagy inhibitor, effectively blocks the phosphorylation of STAT3 and NF-κB, suppresses the infiltration of macrophages and lymphocytes, and suppresses the release of multiple pro-fibrotic cytokines/chemokines	positively regulate
Yeo et al. (Tai et al., 2013)	mouse breast cancer cells	downregulating the autophagy regulator FIP200 in mouse breast cancer models impaired STAT3 or TGFβ/Smad pathway	positively regulate
Maycotte et al. (Su et al., 2014)	breast cancer cells	autophagy can upregulate p-STAT3; when autophagy is inhibited, p-STAT3 is downregulated and tumor cell growth is inhibited in autophagy-dependent breast cancer cell lines	positively regulate
Kang et al. (Qin et al., 2022013)	pancreatic cancer cells	autophagy activated by RAGE promoted IL-6-induced STAT3 activation; in addition, downregulation of autophagic activity in RAGE-targeted knockout KC mice inhibited STAT3 activation and ATP generation in mitochondria and delayed tumor development.	positively regulate

### Regulation of Tumor Autophagy by STAT3

Several studies have shown that STAT3 plays an important role in the regulation of autophagy (Yokoyama et al., 2007; Kroemer et al., 2010; Du Toit, 2012; Shen et al., 2012; Maycotte et al., 2014). Shen *et al.* have found that chemical inhibition of STAT3 induces autophagy, while high STAT3 expression strongly inhibits autophagy both *in vitro* and *in vivo*. Notably, different subcellular localization patterns of STAT3 affect autophagy in various ways. For example, activation of nuclear STAT3 is followed by upregulation of Bcl-2 expression, which disrupts the formation of Beclin 1/Vps34 complex and ultimately inhibits autophagy; in contrast, inactivated STAT3 usually induces upregulation of autophagy (Liu et al., 2018). Cytoplasmic STAT3 inhibits autophagy by binding its SH2 domain to the C-terminal (aa259-552) of protein kinase R (PKR), thereby preventing the phosphorylation of eukaryotic initiation factor 2a (EIF2a), which is necessary for the induction of autophagy initiated by the LC3b and ATG5 cascades (Rouschop et al., 2010). In addition, mitochondrial STAT3 inhibits oxidative stress-induced autophagy and drastically protects mitochondria from mitotic degradation (You et al., 2015a).

Many studies have shown that STAT3 phosphorylation negatively regulates autophagy. Siegelin *et al.* evaluated the *in vitro* and *in vivo* efficacy of sorafenib, a multikinase inhibitor, on glioblastoma cells and found that treatment of patient-derived glioblastoma cells with low-concentration sorafenib significantly inhibited cell proliferation and STAT3 phosphorylation and induced apoptosis and autophagy, resulting in significant inhibition of intracranial glioma growth (Siegelin et al., 2010). You *et al.* found in a study on drug resistance mechanisms in lung cancer that crizotinib induced cytoprotective autophagy by inhibiting STAT3 expression in lung cancer cells, which led to the development of drug resistance (You et al., 2015b). Therefore, when crizotinib is targeted in treating lung cancer patients, inhibition of autophagic activity could improve the efficacy of therapy. Chen *et al.* demonstrated the antagonistic regulation of autophagy by cyclic HIPK3 (circHIPK3) and linear HIPK3 in lung cancer, in which the deletion of cycHIPK3 induced autophagy through the MIR124-3p-STAT3-PRKAA/AMPKa axis and significantly inhibited cell proliferation, migration, and invasion (Chen et al., 2020). In research on hepatocellular carcinoma (HCC), Tai *et al.* found that sorafenib and the novel sorafenib derivative SC-59 activated autophagy and inhibited tumor cell growth in a dose- and time-dependent manner in several HCC cell lines *via* a mechanism that led to disruption of the Beclin 1-Mcl-1 complex through downregulation of p-STAT3, which in turn decreased Mcl-1 expression; however, sorafenib did not affect the amount of Beclin 1 (Tai et al., 2013). Su *et al.* showed that overexpression of STAT3 in HCC cell lines inhibited autophagy; however, after further dephosphorylation of STAT3 using SC-2001, the release of Beclin 1 increased and the level of autophagy increased significantly (Su et al., 2014).

Real *et al.* found in estrogen receptor-negative metastatic breast cancer cell lines that activation of the STAT3-Bcl-2 pathway inhibited the autophagy and apoptosis of these cells, increased their survival benefits, and contributed to the development of drug resistance (Real et al., 2018). Qin *et al.* found that the adding of IL-6 to starvation-induced lymphoma U937 cells significantly increased the phosphorylation level of STAT3, while the level of autophagy was significantly downregulated, suggesting that IL-6 inhibits cellular autophagy through activation of the STAT3 signaling pathway (Qin et al., 2022). Kim *et al.* reported that an herbal formulation SH003 activated autophagy by decreasing the phosphorylation level of STAT3 and could be used as a therapeutic strategy for hypoxia-mediated chemotherapy resistance (Kim et al., 2020). CYT997 is a novel microtubule-disrupting agent that is expected to be an antitumor candidate for gastric cancer treatment. It has been shown to induce autophagy and apoptosis in gastric cancer cells by activating mitochondrial ROS accumulation and silencing the JAK2/STAT3 pathway (Cao et al., 2020). Blessing *et al.* have been looking for drugs that induce autophagy in cancer cells to eliminate residual ovarian cancer cells after conventional surgery and chemotherapy; they found that crizotinib induced autophagy and mediated apoptosis in cancer cells by reducing the phosphorylation of STAT3 and Bcl-2 expression when treating ovarian cancer, which provided a new idea for ovarian cancer treatment (Blessing et al., 2020). Chen *et al.* found that autophagy was induced by knockdown of STAT3 in STK11 mutant lung cancer cell lines (A549 and H838), indicating that circular RNA may be a potential therapeutic target for lung cancer (Chen et al., 2020). In addition to activated STAT3, non-phosphorylated STAT3 in the cytoplasm can also inhibit cellular autophagy (You et al., 2015a). Moreover, in ovarian (Chu et al., 2020), gastric (Nie and Yang, 2017), and colorectal cancers (Zhang et al., 2019), it has been shown that downregulation of p-STAT3 activity increases autophagy of these tumor cells, and the occurrence and progression of these tumors are also suppressed.

However, some studies have also concluded the opposite: STAT3 positively regulates autophagy. Yamada *et al.* found that the activated IL-6/STAT3 pathway upregulated autophagy levels in pancreatic cancer cells (Yamada et al., 2012). Pratt *et al.* found in glioblastoma U87 cells that ConA induced overexpression of MT1-MMP gene and protein, followed by increased STAT3 phosphorylation, which finally led to increased autophagy (upregulated expressions of biomarker BNIP3 gene and protein) (Pratt and Annabi, 2014). According to Yang, under glucose-deficient conditions *in vitro* and *in vivo*, the kinase activator MOB1A, which plays an important role in many diseases and cancers, plays a key role in the development of gallbladder cancer (GBC) by promoting autophagy through activation of the IL6/STAT3 signaling pathway and modulation of gemcitabine chemosensitivity (Yang et al., 2020).

Chemotherapy resistance is one of the main reasons for the low survival rate of tumor patients, and it has been shown that formation of chemoresistance in cancer cells is associated with its altered autophagic activity. Meng *et al.* further showed that STAT3 could promote the transcription of activating transcription factor 6 (ATF6), which induced endoplasmic reticulum (ER) stress to enhance cellular autophagy activity. Finally, the cancer cells became resistant to both cisplatin and paclitaxel treatments. This study revealed that chemoresistance in cancer cells may be mediated through STAT3/ATF6-induced autophagy (Meng et al., 2020). A recent study has shown that prostate cancer stem cells (PCSCs) have an enhanced tendency to be chemoresistant and may be a prognostic factor for prostate cancer recurrence; the activated STAT3 modulates chemoresistance in PCSCs by protecting autophagy and regulating MDR1 on the surface of PCSCs, which sheds new light on the selective targeted therapy against PCSCs (Talukdar et al., 2020).

### Regulation of Autophagy on STAT3

Researches has shown that autophagy mainly shows a positive regulation of STAT3. Inhibition of autophagy by 3-methyladenine (3-MA), a specific autophagy inhibitor, effectively blocks the phosphorylation of STAT3 and NF-κB, suppresses the infiltration of macrophages and lymphocytes, and suppresses the release of multiple pro-fibrotic cytokines/chemokines (Shi et al., 2020). Yeo *et al.* found that downregulating the autophagy regulator FIP200 in mouse breast cancer models impaired STAT3 or TGFβ/Smad pathway, respectively, and diminished the tumor-initiating properties of both ALDH+ and CD29hiCD61 + breast cancer stem cells, thereby limiting tumor growth and reducing the number of breast cancer stem cells (Yeo et al., 2016). According to Maycotte *et al.* (Maycotte et al., 2014; Maycotte et al., 2015) and Romero *et al.* (Romero et al., 2019), different breast cancer cell lines can be either autophagy-dependent or non-autophagy-dependent. For some breast cancer cells, autophagy is essential for survival even in the absence of any nutritional stress. Such a phenotype is even more abundant in triple-negative breast cancer (TNBC) cell lines because the TNBC cell lines are more dependent on STAT3 phosphorylation, whereas autophagy can upregulate p-STAT3; when autophagy is inhibited, p-STAT3 is downregulated and tumor cell growth is inhibited in autophagy-dependent breast cancer cell lines. In contrast, no such phenomenon has been observed in autophagy-independent breast tumors. Therefore, autophagy inhibitors and other drugs may be synergistic when used in combination for autophagy-dependent TNBC, and inhibition of autophagy may be a potential therapeutic strategy for TNBC that currently lacks effective targeted therapy. Similar results have obtained in some studies on pancreatic cancer (Kang et al., 2012; Gong et al., 2014): autophagy activated by RAGE promoted IL-6-induced STAT3 activation; in addition, downregulation of autophagic activity in RAGE-targeted knockout KC mice inhibited STAT3 activation and ATP generation in mitochondria and delayed tumor development.

## Summary and Prospects

As a degradation and reuse process of damaged proteins or organelles in eukaryotic cells, autophagy maintains the intracellular environment stable in response to a range of extracellular damages. However, these damages may trigger STAT3 signaling pathway, another key downstream signaling pathway in the stress responses, which is closely related to autophagy.

It has been shown that STAT3 and autophagy are associated with the development of various tumors. STAT3 negatively regulates autophagy in most studies, although some studies showed the opposite. The different results may be explained by the differences in cell lines and/or STAT3 phosphorylation sites. For example, the phosphorylation site used was Ser727 in a pancreatic cancer experiment (Yamada et al., 2012) but was Tyr705 in the U937 cell experiment (Qin et al., 2022013). Although pancreatic cancer cells were used in both experiments, the phosphorylation site played an important role in the localization of p-STAT3 in mitochondria. In addition, different intercellular localization of STAT3 also has different effects on autophagy. Furthermore, they may form a feedback loop, which may yield different results at different nodes of the assay (Jonchère et al., 2013; Myojin et al., 2019). In addition, autophagy can also influence tumor progression by regulating the phosphorylation level of STAT3, although it has only been investigated in a limited number of studies.

Recent studies have shown that autophagy inhibition has promising clinical applications in cancer therapy. Combination of hydroxychloroquine (an autophagy inhibitor) with temsirolimus (an inhibitor of mammalian target of rapamycin, mTOR) (Rangwala et al., 2014) or bortezomib (a proteasome inhibitor) (Vogl et al., 2014) has been found to be tolerable and effective in phase I clinical trials. Thus, autophagy inhibition may be a powerful complement to STAT3-targeted therapies. However, the overall inhibition of autophagy poses a new problem. That is, normal cells have reduced tolerance to harsh environments. Autophagy is a defense mechanism in normal cells, and activation of autophagy has been reported to protect hepatocytes from chemically-induced hepatotoxicity (Ni et al., 2012). Thus, research and development of more targeted drugs or delivery methods remains a challenge for clinical application.

In summary, STAT3 interacts with autophagy in a very complex manner, involving various factors such as phosphorylation sites, mode of action, and subcellular localization. The specific mechanisms warrant further investigations. A series of targeted drugs exert their antitumor effects by blocking STAT3 signaling, which inevitably affects the autophagic pathway (Xia et al., 2018). Therefore, a clearer understanding of the regulatory mechanisms between STAT3 and autophagy will help the R&D of new drugs targeting the STAT3-autophagic pathway and shed new light on tumor prevention and treatment.

## Data Availability

The original contributions presented in the study are included in the article. Further inquiries can be directed to the corresponding authors.

## Abbreviations

ATG, autophagy related gene; ATF, activating transcription factor; EGFR, epidermal growth factor receptor; GBC, gallbladder cancer; IARC, The international agency for research on cancer; JAK, janus kinase; KDR, kinase-insert domain-containing receptor; MET, mesenchymmal—epithelial transition Factor; MT1-MMP, membrane-type 1 matrix metalloproteinase; mTOR, mammalian target of rapamycin; mPRα, membrane progesterone receptor α; PCSCs, prostate cancer stem cells; PKR, protein kinase R; RTKs, receptor tyrosine kinases; RAGE, receptor for advanced glycation endproducts; RB1CC1, RB1-inducible coiled-coil 1; STAT, signal transducer and activator of transcription; SLS, stone-like structures; TNBC, triple-negative breast cancer; ULK1, unc-51-like kinase 1; VEGF, vascular endothelial growth factor.
